# Unanswered Questions Regarding Sex and BMP/TGF-β Signaling

**DOI:** 10.3390/jdb6020014

**Published:** 2018-06-16

**Authors:** Tapan A. Shah, Melissa B. Rogers

**Affiliations:** Rutgers—New Jersey Medical School, Microbiology, Biochemistry, & Molecular Genetics, Newark, NJ 07103, USA; shaht3@njms.rutgers.edu

**Keywords:** BMP, TGF-β, signaling, sex, chromosomes, XIST, genomic imprinting, hormones, fibrosis

## Abstract

Crosstalk between the BMP and TGF-β signaling pathways regulates many complex developmental processes from the earliest stages of embryogenesis throughout adult life. In many situations, the two signaling pathways act reciprocally. For example, TGF-β signaling is generally pro-fibrotic, whereas BMP signaling is anti-fibrotic and pro-calcific. Sex-specific differences occur in many diseases including cardiovascular pathologies. Differing ratios of fibrosis and calcification in stenotic valves suggests that BMP/TGF-β signaling may vary in men and women. In this review, we focus on the current understanding of the interplay between sex and BMP/TGF-β signaling and pose several unanswered questions.

## 1. Introduction

The distinct developmental mechanisms that bring about the dramatic differences in male and female characteristics are well studied. However, understanding of the impact of sex-associated signaling on the bone morphogenetic protein (BMP), transforming growth factor (TGF)-β and other pathways in developing animals and during adult life is incomplete. Cardiac valvulogenesis is just one of the many complex developmental processes where both BMP and TGF-β signals, along with WNT, fibroblast growth factor (FGF), NOTCH, vascular endothelial growth factor (VEGF) and epidermal growth factor (EGF) signals, orchestrate differentiation and morphology [1,2,3]. As in many adult diseases, processes that control normal embryonic processes also are involved in valve pathologies. Recent reports described dissimilarities in the ratios of fibrotic to calcified tissue in equally stenotic valves from men versus women [4,5,6,7]. Because TGF-β signaling is generally pro-fibrotic and BMP signaling is generally anti-fibrotic, we wondered if sex-associated changes in this balance contributed to the skewed sex distribution of valvular heart disease [4,7,8,9,10,11]. Here, we review the current state of understanding regarding the impact of sex on BMP and TGF-β signaling and identify several unanswered questions. 

## 2. Sex Chromosomes

Most biological discussions of sex start with the X- and Y-chromosomes. Basic mechanisms that may influence BMP and TGF-β signaling would include X- or Y-linked inheritance of variant alleles and differences in X-chromosome inactivation. Of 64 mammalian BMP/TGF-β ligands, receptors, canonical signal mediators and extracellular and intracellular antagonists, only *Bmp15* (GDF9B) maps to the X chromosome in humans, rodents and sheep (Supplemental Table S1 and [12,13,14,15]). BMP15, an oocyte-derived growth and differentiation factor, is essential for folliculogenesis, granulosa cell function and many aspects of female fertility [16]. Interestingly, although clearly essential, mutations of the *Bmp15* gene can have the opposite effects on fecundity in different species [17,18,19]. Such differences in the impact of BMP15 underscore the challenges of translating how signaling pathways crosstalk in model organisms to agriculturally important species and humans.

Of greater overall impact to female biology, both the BMP and TGF-β signaling pathways regulate a key factor in X-chromosome inactivation. This critical process assures similar ratios of X to autosome gene expression between XY males and XX females. In female cells, one of the two X-chromosomes is transcriptionally inactivated by a mechanism involving the noncoding RNA XIST. Six members of the BMP/TGF-β signaling pathway (ACVR1B, BMPR2, SMAD2, SNIP1 and ZFYVE9) were identified in a screen for X-chromosome inactivation modulators [20]. Chromatin immunoprecipitation experiments determined that BMP signaling directly induced the expression of XIST [20]. In contrast, TGF-β1 downregulated XIST in vitro and in vivo. Significantly, TSIX, a negative regulator of XIST, was upregulated upon TGF-β stimulation [21]. Antagonism between the BMP and TGF-β pathways would profoundly influence X-chromosome dosage compensation and thus female biology on a cellular level [20].

Unanswered questions: Human X-chromosome inactivation and reactivation have profound consequences on cellular reprogramming and disease [22]. Likewise, BMP/TGF-β signaling strongly impacts pluripotency and differentiation. How does the upstream modulation of XIST expression by BMP/TGF-β signaling impact the downstream processes affected by these pathways? Does the ratio of BMP/TGF-β signaling affect the 15% of X-linked genes that escape from X-inactivation in human females, many in a tissue-specific pattern [23]? What is the relationship between pathological conditions that alter X-chromosome inactivation and BMP/TGF-β signaling? 

## 3. Genetic Imprinting

Genetic Imprinting is another influential cellular process controlled by sex, in this case, that of the parents. Imprinted genes are expressed on either the maternal or paternal allele, but not both. This monoallelic expression causes a variant or mutated allele to produce a different phenotype based on the parental origin of that imprinted gene. Two hundred and fifty human and 150 mouse imprinted genes were surveyed (Supplemental Table S1 and [24,25]). The ligand BMP8B is predicted to have a paternal genetic imprint in humans. The extracellular antagonist Decorin is maternally imprinted in mouse, but not humans [25]. Decorin binds and sequesters the TGF-β ligand, thereby blocking the ligand-receptor interaction and the downstream signaling transduction [26]. Furthermore, at least two imprinted long noncoding (lnc) RNAs (H19 and MEG3) and their miRNA derivatives (e.g., miR-675-3p and -5p) have been shown to regulate key BMP/TGF-β ligands and signaling intermediaries, including BMP4, SMAD1 and SMAD5 [27,28]. These two lncRNAs are imprinted in both humans and mice and have been shown to influence mesenchymal stem cell lineage decisions such as myogenesis, adipogenesis and osteogenesis that BMP/TGF-β ligands also direct [27,28]. 

Unanswered questions: The epigenetic regulation of imprinted genes controls fetal and postnatal growth, with lifelong metabolic consequences such as obesity that impact health [29]. BMP and TGF-β signaling govern the differentiation of cells into myoblasts, adipocytes, chondrocytes or osteoblasts. How do parentally-imprinted regulators impact critical differentiation choices controlled by BMP/TGF-β signaling?

## 4. Hormones

Beyond the cell-intrinsic impact of the sex chromosomes and imprinting, hormones such as estrogen and androgens are essential drivers of female and male characteristics and function. Furthermore, the natural developmental variation in the hormonal milieu, for example during puberty, pregnancy, lactation and menopause, is substantial. In contrast to X-chromosome inactivation and imprinting, which alter the intrinsic nature of each cell, hormones are extrinsic factors that coordinate cell behaviors on a physiological scale. Unsurprisingly, sex hormones directly regulate many members of the BMP/TGF-β signaling pathways (Table 1). For example, estrogen directly induces *Bmp2* and *Bmp6* transcription [30,31]. In contrast, estrogen inhibits TGF-β signaling by stimulating SMAD2/3 protein degradation [32]. Testosterone was shown to significantly alter the expression of 20 members of the BMP/TGF-β pathway in skeletal muscle progenitors (satellite cells [33]). BMPs and TGF-βs have a long history in reproductive endocrinology. For example, the anti-Müllerian hormone (AMH), also known as the Müllerian-inhibiting substance (MIS), is a member of the superfamily that was discovered in 1947 [34]. An exceedingly complex network of steroid hormone interactions with BMP/TGF-β pathways has been described in reproductive organs. The many highly conserved roles in germ cell-specific and reproductive biology across all animals have been extensively reviewed recently [35,36,37,38]. Estrogen, progesterone and androgens interact with nearly all the members of the TGF-β superfamily (TGF-βs, BMPs, activin, inhibins, anti-Müllerian hormone, growth differentiation factors (GDFs), LEFTY and NODAL) in females [16]. Although possibly more extensively studied in the context of female reproduction, a complex network of BMP/TGF-β signaling is also essential in male reproductive biology [39,40]. 

Unanswered questions: A daunting web of BMP/TGF-β additive, synergistic and antagonistic actions among members of the ligand superfamily and signal mediators occurs in reproductive tissues. Each interaction within this panoply “may” occur in other tissues. The challenge is to identify which interactions also occur in other tissues.

## 5. Crosstalk and Balance

In just the context of cardiovascular biology, sex profoundly influences heart and vascular health, with estrogen generally playing a protective role [51,52]. That a “one size fits all” approach to cardiovascular treatment cannot work for men and women is now widely recognized [52]. Several cardiovascular diseases, including aortic valve stenosis, exhibit sex-specific differential levels of calcified and fibrotic tissues. The reduced blood flow associated with aortic valve stenosis is life-threatening. Unfortunately, treatment is limited to surgical replacement. Both calcification and fibrosis impair valve leaflet mobility. Studies have shown that valves from men typically have greater aortic valve calcification, whereas women have a greater fibrosis score despite equal levels of stenosis and loss of function [5,6,7]. We postulate that the balance of BMP/TGF-β signaling may differ in the valves from males and females. Many members of the TGF-β superfamily, particularly the founding members TGF-β1 and -β2, but also activin A, myostatin and BMP9, promote fibrosis in various tissues [4,53]. Others, for example TGF-β3, BMP2 and BMP7, oppose fibrosis in multiple organs [53,54,55,56,57,58,59,60,61,62,63]. Different ligands often promote alternative lineage choices. For example, TGF-β1 inhibits calcific nodule formation in aortic valves in vivo by inducing SOX9, a pro-chondrogenic, anti-osteogenic transcription factor [64]. On the other hand, BMP2 and its downstream effectors, e.g.*,* phosphorylated SMAD1/5/8(9), are potent pro-osteogenic signals strongly implicated in pathological calcification [65,66,67,68,69,70]. In a few models of organ fibrosis, the antagonistic nature of TGF-β and BMP signaling has been directly observed in the same tissues. In these in vitro and in vivo studies, TGF-β signaling promoted extracellular matrix synthesis and epithelial-mesenchymal transition (EMT), whereas BMP2 negatively regulated these pro-fibrotic processes [54,71,72]. Understanding this interplay between BMP and TGF-β signaling will potentially reveal potential strategies to control fibrotic, calcific and other pathologies [53,73].

Unanswered questions: Clear sex-specific differences in the relative levels of calcification and fibrosis in aortic valves occur. Although BMP/TGF-β signaling strongly influences these processes, few studies have addressed the regulation of these pathways in each sex. What are the relative levels of each BMP/TGF-β ligand in healthy and diseased valves in men and woman? How do sex and hormonal status influence the relative activities of signaling mediators and extracellular and intracellular antagonists of signaling? Most importantly, what therapeutic strategies may modulate the balance of BMP/TGF-β signaling optimally for each sex?

## 6. Concluding Remarks

Many factors lead to sex-specific differences in disease incidences and manifestations and in therapeutic efficacy [74]. These include cell-intrinsic genetic and cell-extrinsic physiological dissimilarities, as well as environmental circumstances such as healthcare inequities [75]. Although increased attention is now paid to social and organismal contrasts between males and females, far less is known regarding the impact of sex on biochemical signaling mechanisms. Despite extensive differences in many diseases, preclinical studies often ignore sex as an important biological variable. Studies often use only male animals or fail to report sex at all. Full understanding of disease processes will only be possible when the effect of sex on signal crosstalk is elucidated. The potential reward will be therapeutic methods to fine-tune the balance of networks involving BMPs, TGF-βs and other signals in both men and women.

## Figures and Tables

**Table 1:** BMP/TGF-β signaling pathway members with molecular evidence of direct regulation by sex-related steroids.

Protein	Steroid	Receptor	Effect (↑ or ↓)	Evidence	Cell or Tissue Type	Reference
**Ligands**						
AMH (MIS)	Estrogen	Estrogen receptor α	↑	Luciferase reporter assay	KK1 cells	[24]
AMH (MIS)	Estrogen	Estrogen receptor β	↓	Luciferase reporter assay	KK1 cells	[24]
BMP2	Estrogen	Estrogen receptor α	↑	Luciferase reporter assay, ovariectomy	C3H10T1/2 cells, bone marrow mesenchymal stem cells	[30,41]
BMP6	Estrogen	Estrogen receptor α	↑	Luciferase reporter assay	MCF-7, T47-D cells, and HepG2 cells	[31]
INHβA (ACTA)	Estrogen	-	↓	Luciferase reporter assay	GRMO2 granulosa cells	[42]
INHβB (ACTB)	Estrogen	-	↓	Luciferase reporter assay	GRMO2 granulosa cells	[42]
TGF-β1	Dihydrotestosterone, R1881 synthetic androgen	Androgen receptor	↑ (PC3mm2 cells), ↓ (primary osteoblasts and LNCaP cells)	Luciferase reporter assay, Chromatin Immunoprecipitation	PC3mm2 cells, LNCaP cells, primary osteoblasts	[43,44,45]
TGF-β3	Estrogen	Estrogen receptor	↑	Chloramphenicol acetyl transferase (CAT) reporter assay	Human MG63 osteosarcoma cells	[46]
**Extracellular Inhibitors**						
Decorin	Progesterone, dienogest synthetic progestin	Progesterone receptor	↑	Chromatin immunoprecipitation	EMOsis cc/TERT and CRL-4003 cells.	[47]
**Receptors**						
TGFβR1 (ALK5)	Estrogen	Estrogen receptor α	↑	Luciferase reporter assay	osteoblasts	[48]
**Intracellular Signal Transducers**						
SMAD3	Dihydrotestosterone	Androgen receptor	↓	Luciferase reporter assay	prostate cancer cell lines	[49]
**Intracellular Inhibitors**						
SMURF1	Mibolerone synthetic androgen	Androgen receptor	↑	Chromatin immunoprecipitation	LNCaP cells	[50]

## Supplementary Materials

The Supplementary Materials are available online at http://www.mdpi.com/2221-3759/6/2/14/s1.

## References

[1] Wu B., Wang Y., Xiao F., Butcher J.T., Yutzey K.E., Zhou B. (2017). Developmental Mechanisms of Aortic Valve Malformation and Disease. Annu. Rev. Physiol..

[2] Kruithof B.P., Duim S.N., Moerkamp A.T., Goumans M.J. (2012). TGFbeta and BMP signaling in cardiac cushion formation: Lessons from mice and chicken. Differentiation.

[3] Dutta P., Lincoln J. (2018). Calcific Aortic Valve Disease: A Developmental Biology Perspective. Curr. Cardiol. Rep..

[4] Sritharen Y., Enriquez-Sarano M., Schaff H.V., Casaclang-Verzosa G., Miller J.D. (2017). Pathophysiology of Aortic Valve Stenosis: Is It Both Fibrocalcific and Sex Specific?. Physiology.

[5] Aggarwal S.R., Clavel M.A., Messika-Zeitoun D., Cueff C., Malouf J., Araoz P.A., Mankad R., Michelena H., Vahanian A., Enriquez-Sarano M. (2013). Sex differences in aortic valve calcification measured by multidetector computed tomography in aortic stenosis. Circ. Cardiovasc. Imaging.

[6] Thaden J.J., Nkomo V.T., Suri R.M., Maleszewski J.J., Soderberg D.J., Clavel M.A., Pislaru S.V., Malouf J.F., Foley T.A., Oh J.K. (2016). Sex-related differences in calcific aortic stenosis: Correlating clinical and echocardiographic characteristics and computed tomography aortic valve calcium score to excised aortic valve weight. Eur. Heart J..

[7] Simard L., Cote N., Dagenais F., Mathieu P., Couture C., Trahan S., Bosse Y., Mohammadi S., Page S., Joubert P. (2017). Sex-Related Discordance Between Aortic Valve Calcification and Hemodynamic Severity of Aortic Stenosis: Is Valvular Fibrosis the Explanation?. Circ. Res..

[8] Andell P., Li X., Martinsson A., Andersson C., Stagmo M., Zoller B., Sundquist K., Smith J.G. (2017). Epidemiology of valvular heart disease in a Swedish nationwide hospital-based register study. Heart.

[9] Kong W.K., Regeer M.V., Ng A.C., McCormack L., Poh K.K., Yeo T.C., Shanks M., Parent S., Enache R., Popescu B.A. (2017). Sex Differences in Phenotypes of Bicuspid Aortic Valve and Aortopathy: Insights From a Large Multicenter, International Registry. Circ. Cardiovasc. Imaging.

[10] Michelena H.I., Mankad S.V. (2017). Sex Differences in Bicuspid Aortic Valve Adults: Who Deserves Our Attention, Men or Women?. Circ. Cardiovasc. Imaging.

[11] Porras A.M., McCoy C.M., Masters K.S. (2017). Calcific Aortic Valve Disease: A Battle of the Sexes. Circ. Res..

[12] Salazar V.S., Gamer L.W., Rosen V. (2016). BMP signalling in skeletal development, disease and repair. Nat. Rev. Endocrinol..

[13] Kanehisa M., Furumichi M., Tanabe M., Sato Y., Morishima K. (2017). KEGG: New perspectives on genomes, pathways, diseases and drugs. Nucleic Acids Res..

[14] Qiagen Ingenuity Pathway Analysis. https://www.qiagenbioinformatics.com/products/ingenuity-pathway-analysis/.

[15] Sanchez-Duffhues G., Hiepen C., Knaus P., Ten Dijke P. (2015). Bone morphogenetic protein signaling in bone homeostasis. Bone.

[16] Ni N., Li Q. (2017). TGFbeta superfamily signaling and uterine decidualization. Reprod. Biol. Endocrinol..

[17] Monsivais D., Matzuk M.M., Pangas S.A. (2017). The TGF-beta Family in the Reproductive Tract. Cold Spring Harb. Perspect. Biol..

[18] Galloway S.M., McNatty K.P., Cambridge L.M., Laitinen M.P., Juengel J.L., Jokiranta T.S., McLaren R.J., Luiro K., Dodds K.G., Montgomery G.W. (2000). Mutations in an oocyte-derived growth factor gene (BMP15) cause increased ovulation rate and infertility in a dosage-sensitive manner. Nat. Genet..

[19] Yan C., Wang P., DeMayo J., DeMayo F.J., Elvin J.A., Carino C., Prasad S.V., Skinner S.S., Dunbar B.S., Dube J.L. (2001). Synergistic roles of bone morphogenetic protein 15 and growth differentiation factor 9 in ovarian function. Mol. Endocrinol..

[20] Sripathy S., Leko V., Adrianse R.L., Loe T., Foss E.J., Dalrymple E., Lao U., Gatbonton-Schwager T., Carter K.T., Payer B. (2017). Screen for reactivation of MeCP2 on the inactive X chromosome identifies the BMP/TGF-beta superfamily as a regulator of XIST expression. Proc. Natl. Acad. Sci. USA.

[21] Zhongzhi W., Masatoshi J., Kayo N., Miho H., Hideo K., Wakana N., Kuniko I., Taiji N., Noritoshi H., Satoshi F. (2016). Long non-coding RNA TSIX is upregulated in scleroderma dermal fibroblasts and controls collagen mRNA stabilization. Exp. Dermatol..

[22] Cantone I., Fisher A.G. (2017). Human X chromosome inactivation and reactivation: Implications for cell reprogramming and disease. Phil. Trans. R. Soc. B.

[23] Disteche C.M., Berletch J.B. (2015). X-chromosome inactivation and escape. J. Genet..

[24] Grynberg M., Pierre A., Rey R., Leclerc A., Arouche N., Hesters L., Catteau-Jonard S., Frydman R., Picard J.Y., Fanchin R. (2012). Differential regulation of ovarian anti-mullerian hormone (AMH) by estradiol through alpha- and beta-estrogen receptors. J. Clin. Endocrinol. Metab..

[25] Monk D., Arnaud P., Apostolidou S., Hills F.A., Kelsey G., Stanier P., Feil R., Moore G.E. (2006). Limited evolutionary conservation of imprinting in the human placenta. Proc. Natl. Acad. Sci. USA.

[26] Hildebrand A., Romaris M., Rasmussen L.M., Heinegard D., Twardzik D.R., Border W.A., Ruoslahti E. (1994). Interaction of the small interstitial proteoglycans biglycan, decorin and fibromodulin with transforming growth factor beta. Biochem. J..

[27] Dey B.K., Pfeifer K., Dutta A. (2014). The H19 long noncoding RNA gives rise to microRNAs miR-675-3p and miR-675-5p to promote skeletal muscle differentiation and regeneration. Genes Dev..

[28] Peng S., Cao L., He S., Zhong Y., Ma H., Zhang Y., Shuai C. (2018). An Overview of Long Noncoding RNAs Involved in Bone Regeneration from Mesenchymal Stem Cells. Stem Cells Int..

[29] Cassidy F.C., Charalambous M. (2018). Genomic imprinting, growth and maternal-fetal interactions. J. Exp. Biol..

[30] Zhou S., Turgeman G., Harris S.E., Leitman D.C., Komm B.S., Bodine P.V., Gazit D. (2003). Estrogens activate bone morphogenetic protein-2 gene transcription in mouse mesenchymal stem cells. Mol. Endocrinol..

[31] Ong D.B., Colley S.M., Norman M.R., Kitazawa S., Tobias J.H. (2004). Transcriptional regulation of a BMP-6 promoter by estrogen receptor alpha. J. Bone Miner. Res..

[32] Ito I., Hanyu A., Wayama M., Goto N., Katsuno Y., Kawasaki S., Nakajima Y., Kajiro M., Komatsu Y., Fujimura A. (2010). Estrogen inhibits transforming growth factor beta signaling by promoting Smad2/3 degradation. J. Biol. Chem..

[33] Braga M., Bhasin S., Jasuja R., Pervin S., Singh R. (2012). Testosterone inhibits transforming growth factor-beta signaling during myogenic differentiation and proliferation of mouse satellite cells: Potential role of follistatin in mediating testosterone action. Mol. Cell. Endocrinol..

[34] Teixeira J., Maheswaran S., Donahoe P.K. (2001). Mullerian inhibiting substance: An instructive developmental hormone with diagnostic and possible therapeutic applications. Endocr. Rev..

[35] Lochab A.K., Extavour C.G. (2017). Bone Morphogenetic Protein (BMP) signaling in animal reproductive system development and function. Dev. Biol..

[36] Wijayarathna R., de Kretser D.M. (2016). Activins in reproductive biology and beyond. Hum. Reprod. Update.

[37] Young J.C., Wakitani S., Loveland K.L. (2015). TGF-beta superfamily signaling in testis formation and early male germline development. Semin. Cell Dev. Biol..

[38] Roly Z.Y., Backhouse B., Cutting A., Tan T.Y., Sinclair A.H., Ayers K.L., Major A.T., Smith C.A. (2018). The cell biology and molecular genetics of Mullerian duct development. Wiley Interdiscip. Rev. Dev. Biol..

[39] Murashima A., Kishigami S., Thomson A., Yamada G. (2015). Androgens and mammalian male reproductive tract development. Biochim. Biophys. Acta.

[40] Ciller I.M., Palanisamy S.K., Ciller U.A., McFarlane J.R. (2016). Postnatal expression of bone morphogenetic proteins and their receptors in the mouse testis. Physiol. Res..

[41] Zhou S., Zilberman Y., Wassermann K., Bain S.D., Sadovsky Y., Gazit D. (2001). Estrogen modulates estrogen receptor alpha and beta expression, osteogenic activity, and apoptosis in mesenchymal stem cells (MSCs) of osteoporotic mice. J. Cell. Biochem..

[42] Kipp J.L., Kilen S.M., Bristol-Gould S., Woodruff T.K., Mayo K.E. (2007). Neonatal exposure to estrogens suppresses activin expression and signaling in the mouse ovary. Endocrinology.

[43] Qi W., Gao S., Wang Z. (2008). Transcriptional regulation of the TGF-beta1 promoter by androgen receptor. Biochem. J..

[44] McCarthy T.L., Centrella M. (2015). Androgen receptor activation integrates complex transcriptional effects in osteoblasts, involving the growth factors TGF-beta and IGF-I, and transcription factor C/EBPdelta. Gene.

[45] Chipuk J.E., Cornelius S.C., Pultz N.J., Jorgensen J.S., Bonham M.J., Kim S.J., Danielpour D. (2002). The androgen receptor represses transforming growth factor-beta signaling through interaction with Smad3. J. Biol. Chem..

[46] Yang N.N., Bryant H.U., Hardikar S., Sato M., Galvin R.J., Glasebrook A.L., Termine J.D. (1996). Estrogen and raloxifene stimulate transforming growth factor-beta 3 gene expression in rat bone: A potential mechanism for estrogen- or raloxifene-mediated bone maintenance. Endocrinology.

[47] Ono Y.J., Terai Y., Tanabe A., Hayashi A., Hayashi M., Yamashita Y., Kyo S., Ohmichi M. (2014). Decorin induced by progesterone plays a crucial role in suppressing endometriosis. J. Endocrinol..

[48] McCarthy T.L., Chang W.Z., Liu Y., Centrella M. (2003). Runx2 integrates estrogen activity in osteoblasts. J. Biol. Chem..

[49] Song K., Wang H., Krebs T.L., Wang B., Kelley T.J., Danielpour D. (2010). DHT selectively reverses Smad3-mediated/TGF-beta-induced responses through transcriptional down-regulation of Smad3 in prostate epithelial cells. Mol. Endocrinol..

[50] Gang X., Wang G., Huang H. (2015). Androgens regulate SMAD ubiquitination regulatory factor-1 expression and prostate cancer cell invasion. Prostate.

[51] Boese A.C., Kim S.C., Yin K.J., Lee J.P., Hamblin M.H. (2017). Sex differences in vascular physiology and pathophysiology: Estrogen and androgen signaling in health and disease. Am. J. Physiol. Heart Circ. Physiol..

[52] den Ruijter H.M., Haitjema S., Asselbergs F.W., Pasterkamp G. (2015). Sex matters to the heart: A special issue dedicated to the impact of sex related differences of cardiovascular diseases. Atherosclerosis.

[53] Walton K.L., Johnson K.E., Harrison C.A. (2017). Targeting TGF-beta Mediated SMAD Signaling for the Prevention of Fibrosis. Front. Pharmacol..

[54] Wang S., Sun A., Li L., Zhao G., Jia J., Wang K., Ge J., Zou Y. (2012). Up-regulation of BMP-2 antagonizes TGF-beta1/ROCK-enhanced cardiac fibrotic signalling through activation of Smurf1/Smad6 complex. J. Cell. Mol. Med..

[55] Izumi M., Masaki M., Hiramoto Y., Sugiyama S., Kuroda T., Terai K., Hori M., Kawase I., Hirota H. (2006). Cross-talk between bone morphogenetic protein 2 and leukemia inhibitory factor through ERK 1/2 and Smad1 in protection against doxorubicin-induced injury of cardiomyocytes. J. Mol. Cell. Cardiol..

[56] Ebelt H., Hillebrand I., Arlt S., Zhang Y., Kostin S., Neuhaus H., Muller-Werdan U., Schwarz E., Werdan K., Braun T. (2013). Treatment with bone morphogenetic protein 2 limits infarct size after myocardial infarction in mice. Shock.

[57] Lepparanta O., Tikkanen J.M., Bespalov M.M., Koli K., Myllarniemi M. (2013). Bone morphogenetic protein-inducer tilorone identified by high-throughput screening is antifibrotic in vivo. Am. J. Respir. Cell Mol. Biol..

[58] Koli K., Myllarniemi M., Vuorinen K., Salmenkivi K., Ryynanen M.J., Kinnula V.L., Keski-Oja J. (2006). Bone morphogenetic protein-4 inhibitor gremlin is overexpressed in idiopathic pulmonary fibrosis. Am. J. Pathol..

[59] Myllarniemi M., Lindholm P., Ryynanen M.J., Kliment C.R., Salmenkivi K., Keski-Oja J., Kinnula V.L., Oury T.D., Koli K. (2008). Gremlin-mediated decrease in bone morphogenetic protein signaling promotes pulmonary fibrosis. Am. J. Respir. Crit. Care Med..

[60] De Langhe E., Cailotto F., De Vooght V., Aznar-Lopez C., Vanoirbeek J.A., Luyten F.P., Lories R.J. (2015). Enhanced endogenous bone morphogenetic protein signaling protects against bleomycin induced pulmonary fibrosis. Respir. Res..

[61] Gao X., Cao Y., Staloch D.A., Gonzales M.A., Aronson J.F., Chao C., Hellmich M.R., Ko T.C. (2014). Bone morphogenetic protein signaling protects against cerulein-induced pancreatic fibrosis. PLoS ONE.

[62] Myllarniemi M., Vuorinen K., Pulkkinen V., Kankaanranta H., Aine T., Salmenkivi K., Keski-Oja J., Koli K., Kinnula V. (2008). Gremlin localization and expression levels partially differentiate idiopathic interstitial pneumonia severity and subtype. J. Pathol..

[63] Farkas L., Farkas D., Gauldie J., Warburton D., Shi W., Kolb M. (2011). Transient overexpression of Gremlin results in epithelial activation and reversible fibrosis in rat lungs. Am. J. Respir. Cell. Mol. Biol..

[64] Huk D.J., Austin B.F., Horne T.E., Hinton R.B., Ray W.C., Heistad D.D., Lincoln J. (2016). Valve Endothelial Cell-Derived Tgfbeta1 Signaling Promotes Nuclear Localization of Sox9 in Interstitial Cells Associated With Attenuated Calcification. Arterioscler. Thromb. Vasc. Biol..

[65] Bostrom K., Watson K.E., Horn S., Wortham C., Herman I.M., Demer L.L. (1993). Bone morphogenetic protein expression in human atherosclerotic lesions. J. Clin. Investig..

[66] Kaden J.J., Bickelhaupt S., Grobholz R., Vahl C.F., Hagl S., Brueckmann M., Haase K.K., Dempfle C.E., Borggrefe M. (2004). Expression of bone sialoprotein and bone morphogenetic protein-2 in calcific aortic stenosis. J. Heart Valve Dis..

[67] Yang X., Meng X., Su X., Mauchley D.C., Ao L., Cleveland J.C., Fullerton D.A. (2009). Bone morphogenic protein 2 induces Runx2 and osteopontin expression in human aortic valve interstitial cells: Role of Smad1 and extracellular signal-regulated kinase 1/2. J. Thorac. Cardiovasc. Surg..

[68] Balachandran K., Sucosky P., Jo H., Yoganathan A.P. (2010). Elevated cyclic stretch induces aortic valve calcification in a bone morphogenic protein-dependent manner. Am. J. Pathol..

[69] Miller J.D., Weiss R.M., Serrano K.M., Castaneda L.E., Brooks R.M., Zimmerman K., Heistad D.D. (2010). Evidence for active regulation of pro-osteogenic signaling in advanced aortic valve disease. Arterioscler. Thromb. Vasc. Biol..

[70] Seya K., Yu Z., Kanemaru K., Daitoku K., Akemoto Y., Shibuya H., Fukuda I., Okumura K., Motomura S., Furukawa K. (2011). Contribution of bone morphogenetic protein-2 to aortic valve calcification in aged rat. J. Pharmacol. Sci..

[71] Flechsig P., Dadrich M., Bickelhaupt S., Jenne J., Hauser K., Timke C., Peschke P., Hahn E.W., Grone H.J., Yingling J. (2012). LY2109761 attenuates radiation-induced pulmonary murine fibrosis via reversal of TGF-beta and BMP-associated proinflammatory and proangiogenic signals. Clin. Cancer Res..

[72] Yang Y.L., Liu Y.S., Chuang L.Y., Guh J.Y., Lee T.C., Liao T.N., Hung M.Y., Chiang T.A. (2009). Bone morphogenetic protein-2 antagonizes renal interstitial fibrosis by promoting catabolism of type I transforming growth factor-beta receptors. Endocrinology.

[73] Akhurst R.J., Hata A. (2012). Targeting the TGFbeta signalling pathway in disease. Nat. Rev. Drug Discov..

[74] Institute of Medicine (2001). Exploring the Biological Contributions to Human Health: Does Sex Matter?.

[75] Shaw L.J., Pepine C.J., Xie J., Mehta P.K., Morris A.A., Dickert N.W., Ferdinand K.C., Gulati M., Reynolds H., Hayes S.N. (2017). Quality and Equitable Health Care Gaps for Women: Attributions to Sex Differences in Cardiovascular Medicine. J. Am. Coll. Cardiol..

