# Supplementary material for: Unanswered Questions Regarding Sex and BMP/TGF-β Signaling
Source: J Dev Biol. 2018 Jun 16;6(2):14. doi: 10.3390/jdb6020014 (PMC6027345; doi:10.3390/jdb6020014)
Supplement: Supplementary file 1 [file jdb-06-00014-s001.pdf]

Supplemental Table S1. Chromosomal locations and genomic imprinting status for members of the BMP/TGF-β signaling pathways.

Protein	HNGC ID	Chromo-some (human/mouse)	Imprinted	Protein	HNGC ID	Chromo-some (human/mouse)	Imprinted
**Ligands**				**Extracellular Inhibitors**			
AMH (MIS)	464	19/10	No	BAMBI	30251	10/18	No
BMP2	1069	20/2	No	BMPER	24154	7/9	No
BMP3	1070	4/5	No	Chordin	1949	3/16	No
BMP3B (GDF10)	4215	10/14	No	DAND5 (Coco)	26780	19/8	No
BMP4	1071	14/14	No	Decorin	2705	12/10	Maternal for mouse, not human (verified [25])
BMP5	1072	6/9	No	Follistatin	3971	5/13	No
BMP6	1073	6/13	No	Gremlin	2001	15/2	No
BMP7 (OP1)	1074	20/2	No	NBL1 (DAN)	7650	1/4	No
BMP8A	21650	1/4	No	LTBP1	6714	2/17	No
BMP8B (OP2)	1075	1/4	Predicted for humans	Noggin	7866	17/11	No
BMP10	20869	2/6	No	Sclerostin	13771	17/11	No
BMP15 (GDF9B)	1068	X/X	No	Twisted Gastrulation	12429	18/17	No
GDF1	4214	19/8	No	**Receptors**			
GDF2 (BMP9)	4217	10/14	No	ACVR2B	174	3/9	No
GDF3	4218	12/6	No	ACVRL1 (ALK1)	175	12/15	No
GDF5 (BMP14)	4220	20/2	No	ACVR1 (ALK2)	171	2/2	No
GDF6 (BMP13)	4221	8/4	No	ALK4 (ACVR1B)	172	12/15	No
GDF9	4224	5/11	No	AMHR2	465	12/15	No

GDF11 (BMP11)	4216	12/10	No	BMPR2	1078	2/1	No
GDF15	30142	19/8	No	BMPRIA (ALK3)	1076	10/14	No
INHα	6065	2/1	No	BMPRIB (ALK6)	1077	4/3	No
INHβA (ACTA)	6066	7/13	No	TGFβR2	11773	3/9	No
INHβB (ACTB)	6067	2/1	No	TGFβR3	11774	1/5	No
INHβC (ACTC)	6068	12/10	No	**Intracellular Signal Transducers**			
INHβE (ACTE)	24029	12/10	No	SMAD1	6767	4/8	No
LEFTYA	3122	1/1	No	SMAD2	6768	18/18	No
LEFTYB	6552	1/1	No	SMAD3	6769	15/9	No
MSTN (GDF8)	4223	2/1	No	SMAD4	6770	18/18	No
NODAL	7865	10/10	No	SMAD5	6771	5/13	No
TGF-β1	11766	19/7	No	SMAD9(8)	6774	13/3	No
TGF-β2	11768	1/1	No	**Intracellular Inhibitors**			
TGF-β3	11769	14/12	No	SMAD6	6772	15/9	No
				SMAD7	6773	18/18	No
				SMURF1	16807	7/5	No
				SMURF2	16809	17/11	No
